# Enhancing the bioconversion of phytosterols to steroidal intermediates by the deficiency of *kasB* in the cell wall synthesis of *Mycobacterium neoaurum*

**DOI:** 10.1186/s12934-020-01335-y

**Published:** 2020-03-30

**Authors:** Liang-Bin Xiong, Hao-Hao Liu, Ming Zhao, Yong-Jun Liu, Lu Song, Zhi-Yong Xie, Yi-Xin Xu, Feng-Qing Wang, Dong-Zhi Wei

**Affiliations:** 1 Shanghai Key Laboratory of Molecular Imaging, Shanghai University of Medicine and Health Sciences, Shanghai, China; 2 State Key Laboratory of Bioreactor Engineering, Newworld Institute of Biotechnology, East China University of Science and Technology, Shanghai, China; 3 College of Pharmacy, Shanghai University of Medicine and Health Sciences, Shanghai, China

**Keywords:** Mycolic acids, Self-enhancement conversion, β-Ketoacyl-acyl carrier protein synthase, Phytosterol, Steroid intermediates

## Abstract

**Background:**

The bioconversion of phytosterols into high value-added steroidal intermediates, including the 9*α*-hydroxy-4-androstene-3,17-dione (9-OHAD) and 22-hydroxy-23,24-bisnorchol-4-ene-3-one (4-HBC), is the cornerstone in steroid pharmaceutical industry. However, the low transportation efficiency of hydrophobic substrates into mycobacterial cells severely limits the transformation. In this study, a robust and stable modification of the cell wall in *M. neoaurum* strain strikingly enhanced the cell permeability for the high production of steroids.

**Results:**

The deletion of the nonessential *kasB*, encoding a β-ketoacyl-acyl carrier protein synthase, led to a disturbed proportion of mycolic acids (MAs), which is one of the most important components in the cell wall of *Mycobacterium neoaurum* ATCC 25795. The determination of cell permeability displayed about two times improvement in the *kasB*-deficient strain than that of the wild type *M. neoaurum*. Thus, the deficiency of *kasB* in the 9-OHAD-producing strain resulted in a significant increase of 137.7% in the yield of 9*α*-hydroxy-4-androstene-3,17-dione (9-OHAD). Ultimately, the 9-OHAD productivity in an industrial used resting cell system was reached 0.1135 g/L/h (10.9 g/L 9-OHAD from 20 g/L phytosterol) and the conversion time was shortened by 33%. In addition, a similar self-enhancement effect (34.5%) was realized in the 22-hydroxy-23,24-bisnorchol-4-ene-3-one (4-HBC) producing strain.

**Conclusions:**

The modification of *kasB* resulted in a meaningful change in the cell wall mycolic acids. Deletion of the *kasB* gene remarkably improved the cell permeability, leading to a self-enhancement of the steroidal intermediate conversion. The results showed a high efficiency and feasibility of this construction strategy.

## Background

Steroidal drugs are the second largest category in the pharmaceutical market. More than 400 kinds of steroid drugs for a wide range of diseases are selling with an annual sale of 100 billion dollars [1]. Modifying the mycobacterial metabolic pathway for accumulating high value-added steroid intermediates [2] is the most important step of the latest upgraded semi-synthetic route in steroidal pharmaceutical industry [3]. By the conversion of low value-added phytosterols, environment friendly extracts from the vegetable oil processing waste, sustainable pine tree bioresource and waste products in papermaking [4], C19 steroids (androst-4-ene-3,17-dione, AD; boldenone, BD; 9α-hydroxy-androst-4-ene-3,17-dione, 9-OHAD) [5, 6] and C22 steroids (22-hydroxy-23,24-bisnorchol-4-ene-3-one, 4-HBC) [7] can be respectively accumulated. Then, almost all kinds of steroid drugs, including adrenocortical and progestational hormones, can be produced by the combinational chemical modifications [8]. For instance, 9-OHAD is a core intermediate and has been used as a cost-effective precursor to synthesize C21 adrenocortical hormone drugs [8]. However, the unsatisfying yield and productivity of the currently used strains has prompted researchers to intensively investigate more efficient and stable strategies for the biosynthesis of important steroidal intermediates [9, 10].

Sterols can be catabolized as the sole carbon and energy source for maintaining the balance of basic physiological metabolism in mycobacteria [9]. The uptake of sterols in cells may be divided into two distinguished stages: (I) the mass transfer stage of sterol molecules and particles to cell surface and (II) the diffusion stage of sterols across the cell wall and membrane. Stage I is mainly depends on the direct contact with the substrates dispersed in the extracellular environment. Early studies on material transfers demonstrated that in the presence of hydroxypropyl-β-cyclodextrin [11], the use of biocompatible water-immiscible organic phase [12] could largely improve the solubilization of sterol substrates in the transformation system. As a result, the cells contacted with the sterols more efficiently. The substrate transfer was enhanced and the conversion productivity was increased accordingly. In addition, the β-cyclodextrin possibly improved the permeability due to the alteration of mycobacterial cell wall structure [13]. Thus, with the addition of glycine and vancomycin, which were inhibitors to the synthesis of mycobacterial cell wall, the cell permeability displayed a marked improvement [14]. However, these strategies employing massive additives are seldom used in the industrial process because of the high costs and low effects. It is noteworthy that most of the aforementioned methods possibly lead to some defects of the cell wall. The mycobacteria cell wall contains extremely rich mycolic acids [15]. This component accounts for 40–60% of the cell dry weight and are probably responsible for the crucial cell permeability characteristic [16, 17]. Rational modifications of the mycolic acid biosynthesis pathway might be reasonable ways to alter the permeability performance of the steroidal conversion microbial cell factories.

**Fig. 1:**
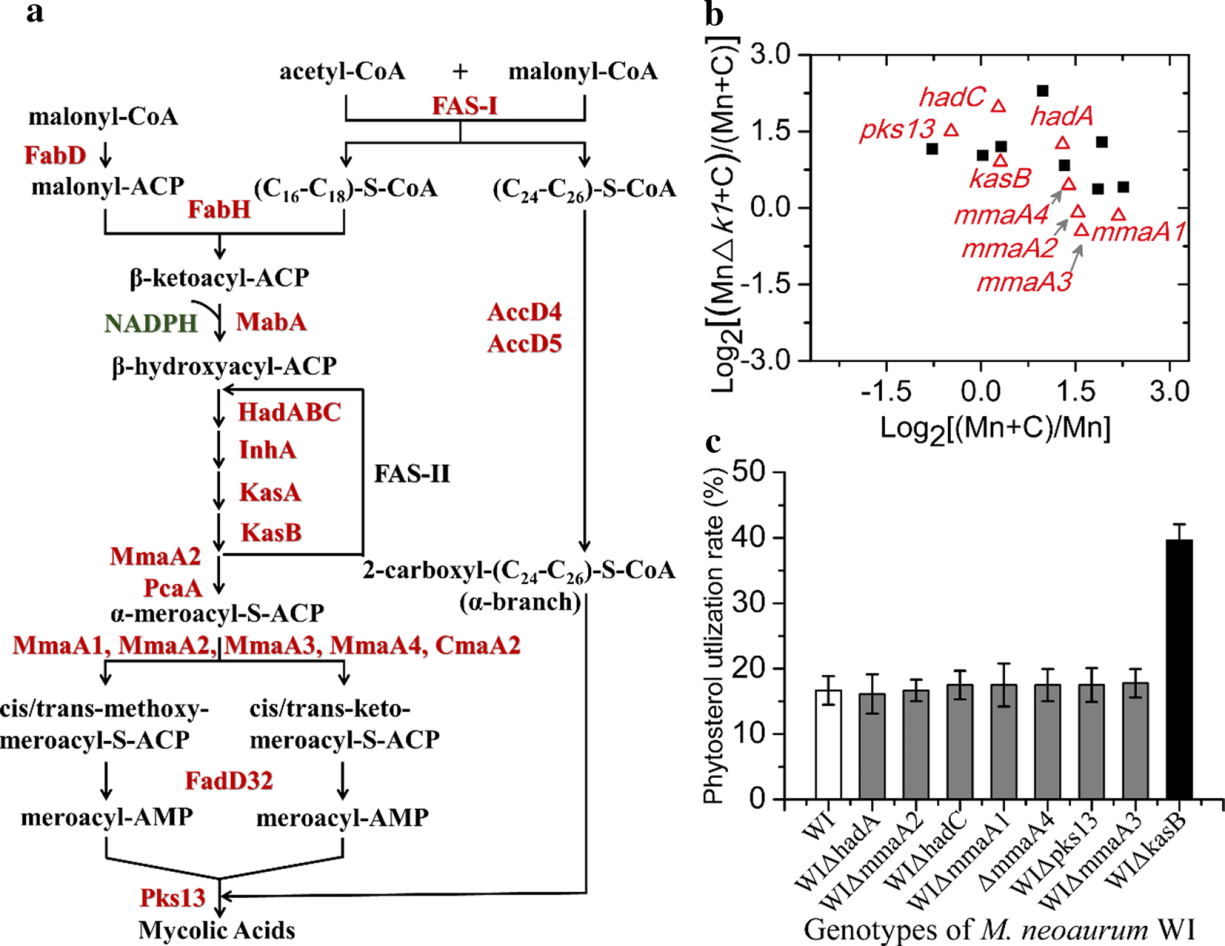
Rational disruption of the mycolic acid synthesis disturbed the sterol conversion. **a** Profile of the mycolic acid synthesis pathway in mycobacteria cells [18]. FAS-I, fatty acid synthase I; FabD, malonyl CoA-acyl carrier protein (ACP) transacylase; FabH, β-ketoacyl-ACP synthase III; MabA, β-ketoacyl-ACP reductase; HadABC, β-hydroxyacyl-ACP dehydratase subunits A, B and C; InhA, enoyl-ACP reductase; KasA, β-ketoacyl-ACP synthase 1; KasB, β-ketoacyl-ACP synthase 2; PcaA, proximal cyclopropanation of alpha-MAs enzyme; MmaA1-4, methyl mycolic acid synthase; CmaA2, cyclopropyl mycolic acid synthase; AccD4, propanoyl-CoA carbon dioxide ligase; AccD5, propionyl-CoA carboxylase; FadD32, long-chain-fatty-acid-AMP synthetase, Pks13, polyketide synthase. **b** Transcription changes in the dispensable genes involved in mycolic acid synthesis. All data indicate log2 fold change ratio of the gene expression. Mn, the wild type *M. neoaurum* was cultured in MYC/02 medium. Mn + C, the wild type strain was cultivated in the presence of phytosterol. MnΔ*kstD1 *+ C, the primary 9-OHAD-producing strain MnΔ*kstD1* was cultured in MYC/02 medium with phytosterol addition. Data were from two independent analyzes. **c** The alternation of sterol utilization rate caused by the targeted gene deletion in 72 h sample time. Data represent the mean standard deviation of three measurements

Mycolic acids are synthesized originally from acetyl-CoA and malonyl-CoA [18]. The C16–C18 and C24–C26 α-alkyl chain is elongated based on Claisen condensation catalyzed by the fatty acid synthase I (FAS-I). The resulting short chain is synthesized by β-ketoacyl-ACP synthases (FabH) to form β-ketoacyl-ACP. Then, a long mero chain can be obtained by the repetitive reductive cycles due to the catalysis of multienzyme fatty acid synthase II complex (FAS-II). Additional elongation cycles are subsequently catalyzed by the two β-ketoacyl-ACP synthase KasA and KasB. After the mero-chain and α-chain are coupled together by the acyl-AMP ligase FadD32 and the polyketide synthase Pks13 and then deoxidized by the mycolate reductase CmrA, the mature mycolate (trehalose monomycolate, TMM) can be synthetized in the mycobacterial cytoplasm. Next, the TMM is transported to the cell periplasm and participates in the subsequent assembly of mycolic acid-related structures, including the polar TDM and mycolic acid methyl esters in the core mycolyl-arabinogalactan-peptidoglycan (MAMEs-AG-PG) complex of cell wall [17]. The FAS-I synthesis gene *fas* is required in *M. smegmatis* [19] and *M. tuberculosis* [20] and the fatty acid synthase II (FASII) enzymes InhA [21], MabA [22], HadB [23], and KasA [24] are also required. The inactivation of these indispensable genes could lead to the lysis of mycobacterial cells [21–24]. The disruption of nonessential genes possibly caused some stable defects only in the cell wall. Thus, the loss of the dispensable genes, such as *hadA*, *hadC* and *kasB* in the mero-mycolic acid synthesis pathway, are worth investigation in the model steroid transformation cells (Fig. 1a) [16, 18].

The biotransformation process is a rate-limiting step in the microbes producing steroid intermediates. It usually takes 120 to 144 h to realize a satisfactory conversion rate of the substrate to target steroid intermediates in the microbes [5, 6, 25]. However, it only takes about 48 to 72 h in most of other prokaryotic microorganisms [26–28]. The long conversion time is primarily attributed to the low permeability of sterol substrates into the cell wall [2]. Promoting the substrate to enter microbial cells by modifying the cell wall may shorten the time required by the bioconversion process and improve the integral production capacity of mycobacterial cells.

Increasing the sterol biotransformation efficiency in *M. neoaurum* through a systemic cell wall engineering technique was rarely reported [2]. The disruption of the genes involved in mycolic acid synthesis in mycobacterial cells was not directly assessed. In the study, the annotated nonessential mycolic acid synthetic genes were inactivated individually. The modification which significantly altered the sterol conversion was further investigated. The result revealed the roles of accessory genes in the formation of mycolic acids and provided an alternative evolution strategy for the microbial transformation of steroidal intermediates.

## Methods

### Strains, plasmids and primers

All strains used in this study are described below (Table 1). *Escherichia coli* DH5α (TIANGEN Biotech. Co., Ltd., Shanghai, China) was used for plasmid amplification. The wild type *M. neoaurum* ATCC 25795 (Mn) was purchased from American Type Culture Collection (ATCC). The C19 steroidal intermediate 9-OHAD producers MnΔ*kstD1* and MnΔ*kstD1*Δ*kstD2*Δ*kstD3* (WI) were constructed by Kang Yao [6]. The C22 steroidal intermediate 4-HBC-producing strain MnΔ*kshA*Δ*hsd4A*Δ*kstD1*Δ*kstD2*Δ*kstD3* (WIII) was constructed by Xu [7]. Others were all derived from the above three *M. neoaurum* strains. Common plasmids (Additional file 1: Table S1) and primers (Additional file 1: Table S2) were used for constructing the mutants.

**Table 1:** Strains used in this study

Name	Description	Source
*E. coli* DH5α	*E. coli* strain for cloning	TIANGEN CO., LTD.
*M. neoaurum* ATCC 25795 (Mn)	Wild type strain, the starting strain	ATCC
MnΔ*kstD1*	*kstD1* deleted in *M. neoaurum* ATCC 25795	[6]
WI	*kstD1*, *kstD2* and *kstD3* deleted in *M. neoaurum* ATCC 25795, 9-OHAD producing strain	[6]
WIΔ*hadA*	*hadA* deleted in WI strain	This study
WIΔ*hadC*	*hadC* deleted in WI strain	This study
WIΔ*kasB*	*kasB* deleted in WI strain	This study
WIΔ*mmaAN*	*mmaAN* (N represents 1, 2, 3, 4) deleted in WI strain	This study
WIΔ*pks13*	*pks13* deleted in WI strain	This study
MnΔ*kasB*	*kasB* deleted in *M. neoaurum* ATCC 25795	This study
MnΔ*kasB *+ *kasB*	*kasB* complemented in MnΔ*kasB* strain	This study
WIII	*kshA1*, *kshA2*, *hsd4A*, *kstD1, kstD2* and *kstD3* deleted in *M. neoaurum* ATCC 25795, 4-HBC producing strain	[7]
WIIIΔ*kasB*	*kasB* deleted in WIII strain	This study

### Media and culture conditions

Media and culture conditions were the same as the previously described conditions [2, 29]. *E. coli* DH5α was inoculated at 37 °C in 5 mL of Luria–Bertani (LB) medium. Kanamycin (50 mg/L) or hygromycin (100 mg/L) was added to the culture medium as required. Mycobacterial strains were firstly cultivated in 5 mL of LB until OD_600_ was between 1.2 and 1.8. Then, according to an inoculum volume ratio of 1:10 (v/v), the cell suspension was inoculated into 30 mL of MYC/01 medium (20.0 g/L glycerol, 2.0 g/L citric acid, 2.0 g/L NH_4_NO_3_, 0.5 g/L K_2_HPO_4_, 0.5 g/L MgSO_4_·7H_2_O, and 0.05 g/L ammonium ferric citrate, pH 7.5) in 250-mL flasks to obtain the mycobacterial seed suspension (OD600 = 1.2–1.8).

For phenotypic identification, according to an inoculum volume ratio of 1:10 (v/v), the cultivated cells were then transferred into 30 mL of minimal medium (MM) (2.0 g/L NH_4_NO_3_, 0.5 g/L K_2_HPO_4_, 0.5 g/L MgSO_4_·7H_2_O, and 0.05 g/L ammonium ferric citrate) with 1 g/L glycerol or 1 g/L cholesterol (purity > 95.0%, Adamas Reagent, Ltd., Shanghai, China). Cells were harvested by the centrifugation at 4000*g* for 10 min.

For the bioconversion in growth cells, according to an inoculum volume ratio of 1:10 (v/v), the cultivated seed cells were inoculated into 30 mL of MYC/02 medium (10.0 g/L glucose, 2.0 g/L citric acid, 2.0 g/L NH_4_NO_3_, 0.5 g/L MgSO_4_·7H_2_O, and 0.05 g/L ferric ammonium citrate, pH 7.5) with 5 g/L phytosterols (purity > 95.0%, every 100 g of phytosterol contained 47.5 g of β-sitosterol, 26.4 g of campesterol, 17.7 g of stigmasterol, 3.6 g of brassicasterol and 4.8 g of undetermined components) (Zhejiang Davi Pharmaceutical Co., Ltd., Zhejiang, China) [29]. Cholesterol (100.0 g/L) and phytosterol (100.0 g/L) was emulsified in Tween 80 (5% w/v) aqueous solution at 121 °C for 60 min before use. The shake flask experiments of *M. neoaurum* strain were carried out at 30 °C and 200 rpm.

For resting cell conversion, according to an inoculum volume ratio of 1:10 (v/v), the cultivated cells were transferred into 150 mL of MYC/02 medium in 1000-mL shake flasks for the growth at 30 °C and 200 rpm. The cells were harvested by the centrifugation at 8000*g* for 15 min, washed with 20 mM KH_2_PO_4_, and diluted into 200 g/L of cell suspensions. The subsequent conversion step was performed in 250-mL flasks containing 100 g/L mycobacterial cells, 20 g/L phytosterols and 80 g/L hydroxypropyl-β-cyclodextrin (HP-β-CD, RSC Chemical Industries Co., Ltd., Jiangsu, China) in at 30 °C and 200 rpm [30]. Standard 9-OHAD (99%) was purchased from J&K Scientific Ltd. (Beijing, China). Standard reference 4-HBC (97%) was purified and identified by ourselves [7].

### Construction of genetically modified strains

Target gene-deleted strains were obtained through allelic homologous recombination in mycobacteria as previously described [31]. p2NIL and pGOAL19 were used for the construction of the homologous recombination plasmids (Additional file 1: Table S1). The knockout-plasmids p19-gene, including p19-*hadA*, p19-*mmaA2*, p19-*hadC*, p19-*mmaA1*, p19-*mmaA3*, p19-*mmaA4*, p19-*pks13* and p19-*kasB*, was transferred into mycobacterial cells via electroporation, respectively. Then, the target gene deficient strain can be obtained following the two-step screening process [32].

To complement the deficient-gene function, the complete gene sequence of *kasB* was firstly amplified from the wild type strain with the primer pairs (C-*kasB*-F & C-*kasB*-R) (Additional file 1: Table S2). After double digestion with EcoRI and HindIII, the enzyme-digested fragment was inserted into the pMV261 to create a recombinant p261-*kasB* plasmid. This constructed recombination plasmid could be used to overexpress the carried *kasB* in multiple copies. Moreover, the expression cassette of the target *kasB* containing a heat shock promoter *hsp60* was obtained from the recombinant p261-*kasB* through double-digestion with XbaI and HindIII then integrated into the pMV306 to create a complemental plasmid p306-*kasB*. The constructed plasmid could be integrated into chromosomal DNA in single copy to complement the disrupted gene function.

### Analysis of cell permeability and steroid uptake performance

The permeability change of cell envelope was estimated by measuring the fluorescence intensity of cells labeled by fluorescein diacetate (FDA, Aladdin Reagents (Shanghai) Co., Ltd., Shanghai, China) according to previous procedures with some minor amendments [33]. The same wet weight of mycobacterial cells were suspended in 4.5 mL of phosphate buffer (cell density reached 10^6^ cells/mL), mixed with 0.5 mL of FDA acetone solution (2 mg/mL) and then vibrated at 32 °C for 10 min before the detection with a Fluoroskan Ascent fluorescence spectrophotometer (Thermo Labsystems Inc., PA, USA). Maximum excitation wavelength for the detection was 485 nm, and the emission wavelength was 538 nm.

The quantity of cholest-4-en-3-one (purity > 95.0%, Shanghai TITAN Scientific Co., Ltd., China) entering mycobacterial cells per unit time was determined to check for the cell permeability change. This steroid was emulsified in Tween 80 (5% w/v) aqueous solution at 121 °C for 60 min in advance for use. The cultivated cells were inoculated into 30 mL of MYC/02 medium with 1.0 g/L cholest-4-en-3-one. After 12-h growth, 5 mL of culture solution was sampled, centrifuged at 12,000*g* for 10 min, washed with 1.0 mL of ddH_2_O for two times, and then washed with 1.0 mL of the mixture of petroleum ether and ethyl acetate (6:4, v/v) to remove the cholest-4-en-3-one from the media. The cells (50 mg, wet weight) were then suspended in 1.0 mL of the mixture of acetonitrile and ddH_2_O (7:3, v/v). Then, 0.8 g of glass beads were added in the suspension. The cells were destroyed with FastPrep-24 instrument (MP Biomedicals, CA, USA) and centrifuged at 12,000*g* for 10 min. Cholest-4-en-3-one entering cells could be released and dissolved in acetonitrile. The extracts were analyzed with a reversed-phase C18-column (250 mm × 4.6 mm) at 254 nm with the Agilent 1100 series HPLC system. The mixture of methanol and water (8:2, v/v) was used as the mobile phase.

### Analysis of mycolic acid methyl esters (MAMEs)

The MAMEs were extracted and analyzed as previously described [2, 17, 34]. Briefly, 50 mg (in wet weight) of mycobacterial cells were collected at 12,000*g* for 10 min. After adding 0.5 mL of the mixture of methanol and chloroform (2:1, v/v), the homogenized mixture was incubated at 60 °C for 2 h and centrifuged at 12,000*g* for 10 min. The polar lipids including TMM and TDM were dissolved in the supernatant.

Next, 500 μL of 10% tetrabutylammonium hydroxide (Sigma-Aldrich LLC., MO, USA) was added to the above defatted cells or 50 mg of whole cells and heated at 100 °C overnight. After cooling, 500 μL of ddH_2_O, 250 μL of dichloromethane, and 62.5 μL of iodomethane (Sigma-Aldrich LLC., MO, USA) were added into the mixture. Then, the diluted mixture was stirred for 30 min and centrifuged at 12,000*g* for 10 min to remove the upper layer. The lower organic layer was washed with 1.0 mL of 1 M hydrochloric acid, followed by 1.0 mL of ddH_2_O. The reaction solution was dried under a stream of nitrogen. The residue was dissolved in a mixture of toluene (0.2 mL) and acetonitrile (0.1 mL), followed by the addition of acetonitrile (0.2 mL) for 1-h incubation at 4 °C. The MAMEs were centrifuged at 12,000*g* for 10 min and then re-suspended in 200 μL of dichlormethane.

The extracted mycolic acids were analyzed by silica gel TLC plates in a solvent system (chloroform: methanol, 90:10, v/v). The mean grayscale intensity of spots in the TLC plate was analyzed with Quantity One (Version 4.6.6, Bio-Rad Laboratories, CA, USA) The relative abundances of the polar mycolic acids (TMM and TDM) and MAMEs were calculated, respectively. The keto-MA spots on preparative silica gel TLC were purified for MALDI-TOF–MS (Xevo G2, Waters, Ltd., MA) analysis as described [16].

### Sterol bioconversion and the extraction and analysis of steroidal intermediates

Both vegetative cells and resting cells were determined to assess the sterol conversion capability [2, 30]. Firstly, the vegetative cell biotransformation medium (0.5 mL) was extracted with the same volume of ethyl acetate. Then the sample containing steroidal intermediates from resting cell transformation system was extracted with ten times of volume of ethyl acetate.

A gas chromatography (GC) system 7820A (Agilent Technologies, CA, USA) was used for the quantitative determination of cholesterol and phytosterols. The ethyl acetate extracts (5 μL) were injected into a DB-5 column (30 m × 0.25 mm (i.d.) × 0.25 μm film thickness, Agilent Technologies, CA, USA). The oven temperature was programmed as follows: 200 °C for 2 min, 200 °C to 280 °C within 4 min, 280 °C for 2 min, 280 °C to 305 °C within 1.5 min, and 305 °C for 10 min. Inlet and flame-ionization detector temperatures were maintained at 320 °C. Nitrogen carrier gas flow was 2 mL/min at 50 °C. The sum of three major components (*β*-sitosterol, campesterol and stigmasterol) was calculated to assess the utilization of phytosterols as previously described [29].

A 1100 series high-performance liquid chromatography system (HPLC) (Agilent Technologies, CA, USA) was employed to analyze the extracts containing 9-OHAD or 4-HBC. The prepared samples were analyzed with a reversed-phase XDB-C18-column (250 mm × 4.6 mm, 30 °C) (Agilent Technologies, CA, USA) at 254 nm. The mixture of methanol and water (8:2, v/v) was used as the mobile phase. The mass concentration of 9-OHAD was calculated using the standard calibration curve constructed at the same time. The mass concentration of 4-HBC produced by the WIII and WIIIΔ*kasB* strain was calculated using the 4-HBC standard calibration curve.

## Results and discussion

### Disruption of the mycolic acid synthesis genes disturbed the sterol conversion

Mycolic acids, as the main cell wall constituent, are generally synthesized in the cytoplasm (Fig. 1a) [17, 18]. The interference with the nonessential gene, such as the (3R)-hydroxyacyl-ACP dehydratase *hadA* and methyl mycolic acid synthase 1 *mmaA1,* etc., involved in the synthesis of mycolic acids might reduce the tightness of the cell wall and lead to a stable change in cell permeability. For further studies, the genes involved in the synthesis of mycolic acids were preliminarily evaluated by the comparative transcriptome analysis between the wild type strain and its primary derivative 9-OHAD-producing strain (MnΔ*kstD1*) [31]. We planned to screen some genes whose transcription levels were remarkably fluctuated during the accumulation of 9-OHAD. However, the transcriptional levels of most of the annotated genes showed discrete variations in the bioconversion of sterols to 9-OHAD (Fig. 1b, Additional file 1: Table S3).

Next, we had to randomly select some dispensable genes and obtained the targeted deletion of the mycolic acid synthesis pathway in the final 9-OHAD-producing strain WI. Interestingly, the inactivation of most of the accessary genes resulted in a slight alteration of sterol utilization rate in all the strains except the WIΔ*kasB* strain (Fig. 1c). As expected, the deletion of the gene remarkably increased the sterol utilization by 143% at the 72-h sampling time. Early studies demonstrated that the *kasB* was a nonessential gene responsible for the extension to full-length mero-mycolic acids in *M. tuberculosis* [16]. The result indicated that a meaningful permeability change might occur in the mutant strain.

### Functional KasB maintained the cell permeability and the balance of steroid uptake in *M. neoaurum*

**Fig. 2:**
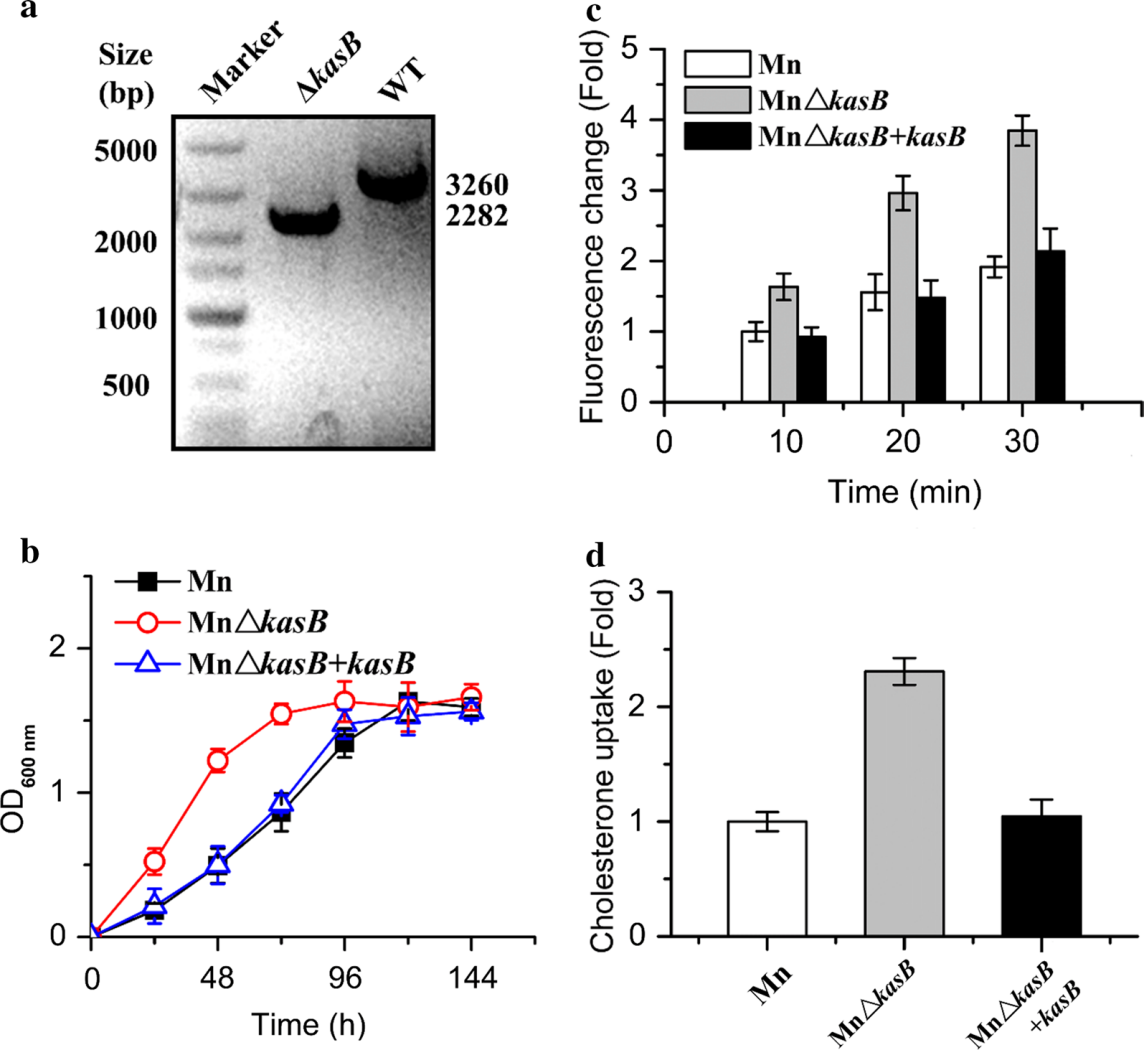
Effects of the deficiency of *kasB* on the cell permeability. **a** Validation of allelic replacement at the *kasB* locus in *M. neoaurum* ATCC 25795. The wild type (WT) 3260-bp was replaced by a 2282-bp fragment ligate with the upstream and downstream homologous arm. **b** Growth characteristic of the *kasB* mutant strain. The wide-type *M. neoaurum* (Mn), the *kasB*-deficient strain (MnΔ*kasB*) and the *kasB*-complemented strain (MnΔ*kasB *+ *kasB*) were cultured in MM containing 1.0 g/L cholesterol. **c** Determination of the cell permeability in the *kasB* mutant strain. The cells were stained with FDA, incubated at 32 °C for 10 min, and analyzed by a fluorescence spectrophotometer. The mutant strain MnΔ*kasB* displayed about two times penetrated FDA compared with that in its parental Mn strain after 30 min of incubation. **d** Influences of the deficiency of *kasB* on the steroid (cholest-4-en-3-one) uptake. The cholest-4-en-3-one entering the cells after 12-h growth in MM containing 1.0 g/L cholest-4-en-3-one was determined. The uptake of cholest-4-en-3-one in the strain MnΔ*kasB* showed about 2.3 times improvement than that of the wild type Mn strain

The possible *kasB* genome region in *M. neoaurum* ATCC 25795 (GenBank Accession No. NZ_JMDW00000000.1) was re-confirmed by comparing the homologous regions in *Mycobacterium tuberculosis* H37Rv (GenBank Accession No. NC_000962), *Mycobacterium smegmatis* mc2 155 (GenBank Accession No. NC_008596) and *Mycobacterium neoaurum* VKM Ac-1815D (GenBank Accession No. CP006936.2). The *kasB* gene (GeneBank: NZ_JMDW01000013.1; Region: 177334…178587, 1254-bp) in *M. neoaurum* shared high sequence identity with its homologs (Additional file 2: Figure S1), indicating its conserved function in mycobacteria. In addition, the flanking genes of *kasB* also had the similar frame. These results proved that the annotation and position of the *kasB* gene was correct (Additional file 2: Figure S1; Additional file 1: Table S4). The allelic homologous recombination was employed to delete the *kasB* cassette in the wild type *M. neoaurum*. A 1171-bp upstream sequence and 1111-bp downstream sequence were amplified to construct the plasmid vector for gene knockout (Additional file 2: Figure S2). PCR and electrophoresis analysis results of the *kasB* region in genomic DNA confirmed the occurrence of allelic replacement in *M. neoaurum* (Fig. 2a).

In mycobacteria, *kasA* and *kasB* encode two distinct fatty acid synthase II complexes. KasA is responsible for the initial elongation of mycolic acids less than 40 carbons, whereas KasB is involved in the extension from 40 carbons to 54 carbons [18]. The subsequent deletion of *kasB* in the mutant strain WI might be disadvantage to test the phenotype. In order to assess the effect of *kasB* on the cell permeability, the MnΔ*kasB* mutant strain and the complemented strain MnΔ*kasB *+ *kasB* were generated for subsequent experiments. The deletion of *kasB* led to an obvious alteration of cell growth in the presence of cholesterol and the MnΔ*kasB* strain growth was much faster than that of its parental wild type strain and the complemented strain (Fig. 2b). The acceleration in growth rate of the MnΔ*kasB* strain was similarly to the result of *mmpL3* deletion in *M. neoaurum* [2]. The enhanced cell permeability might raise the supplement of steroids in the cell wall deficient strain. Subsequently, the permeability of *kasB*-deficient strain was assessed through determining the fluorescence intensity of the cells after labeling with fluorescein diacetate (FDA) (Fig. 2c). The result showed that the MnΔ*kasB* mutant strain had the more permeable cell wall than that of the wild type strain. The penetrated FDA of MnΔ*kasB* strain was about two times compared to the parental Mn strain after 30 min of incubation. This wild type property could be restored in the mutant strain upon the introduction of the complete functional *kasB* gene. To further confirm this, the analog of cholesterol, cholest-4-en-3-one was employed as a label to check for the cell permeability to steroids [2]. The analysis indicated that the improved the cell wall permeability indeed resulted in about 2.3 times enhancement in the uptake of cholest-4-en-3-one in the *kasB*-deficient strain after 12 h of growth (Fig. 2d). The improvement might be interpreted as a chain effect caused by the enhanced cell permeability. These results further confirmed that the observed enhancement of sterol conversion and utilization was probably attributed to the improved cell permeability through the inactivation of *kasB* function.

### Deletion of *kasB* changed the composition of cell wall mycolic acids

Previous studies demonstrated that *kasB* was dispensable for normal mycobacterial growth in *M. marinum* and *M. smegmatis* [24, 35]. The *kasB* in *M. neoaurum* was proved to play a similar role in mycobacterial growth. The mechanism for the alternation of cell permeability with respect to the *kasB* deficiency in *M. neoaurum* remains unclear. Notably, KasB is responsible for the extension of mero-mycolic acid carbon chain [16]. This function indicated that the increased permeability was likely attributed to the changed KasB-responsible cell wall mycolic acid synthesis in the mutant strain.

**Fig. 3:**
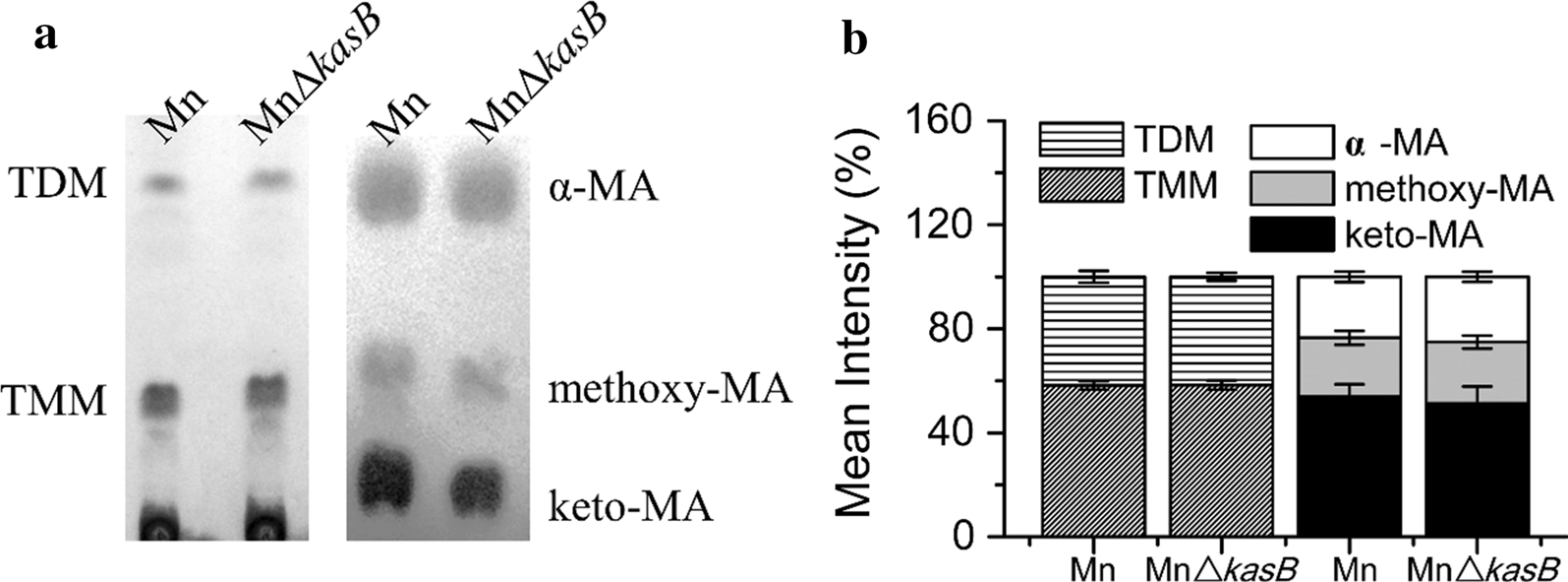
Effects of *kasB* on the component of cell wall mycolic acids in *M. neoaurum*. **a** The strain carrying the wild type *kasB* (Mn) or the deficient *kasB* (MnΔ*kasB*) was cultivated in the presence of 1.0 g/L phytosterols. MAMEs (α-, methoxy- and keto- forms of mycolic acids) were isolated from *M. neoaurum* cells. TLC plates were revealed with cupric sulfate (10% w/v in an 8% v/v phosphoric acid solution). **b** Relative intensity of the mycolate compared to the total mycolates was calculated. The deletion of *kasB* caused a slight disturbance of MAMEs components in MnΔ*kasB* (α-: 25.1%, methoxy-: 23.5%, and keto-: 51.4%) compared with that of the Mn strain (α-: 23.5%, methoxy-: 22.6%, and keto-: 53.9%)

In the TLC analysis results, the polar TMM and TDM showed no obvious difference, whereas the mycolic acid methyl esters (MAMEs) displayed a slight decrease in the MnΔ*kasB* mutant strain (Fig. 3a; Additional file 2: Figure S3). The relative abundances of the α-MA, methoxy-MA and keto-MA were respectively 25.1%, 23.5%, and 51.4% in the MnΔ*kasB* strain and 23.5%, 22.6%, and 53.9% in its parental strain Mn (Fig. 3b). The decrease in keto-MA content was similar to the trend of the *kasB*-deleted *M. tuberculosis* [16]. Next, the keto-MA spot was purified and analyzed by MALDI-TOF MS. The spectrogram showed a changed keto-MA in MnΔ*kasB* strain compared with that of the wild type Mn strain (Additional file 2: Figure S4). Considering the function of *kasB* in other mycobacteria, the inactivation of the *kasB* was most likely shortened the length of the keto-MA, the specific changes of MA need to be further determined.

### Loss of *kasB* led to a remarkable improvement in steroid intermediate productivity

**Fig. 4:**
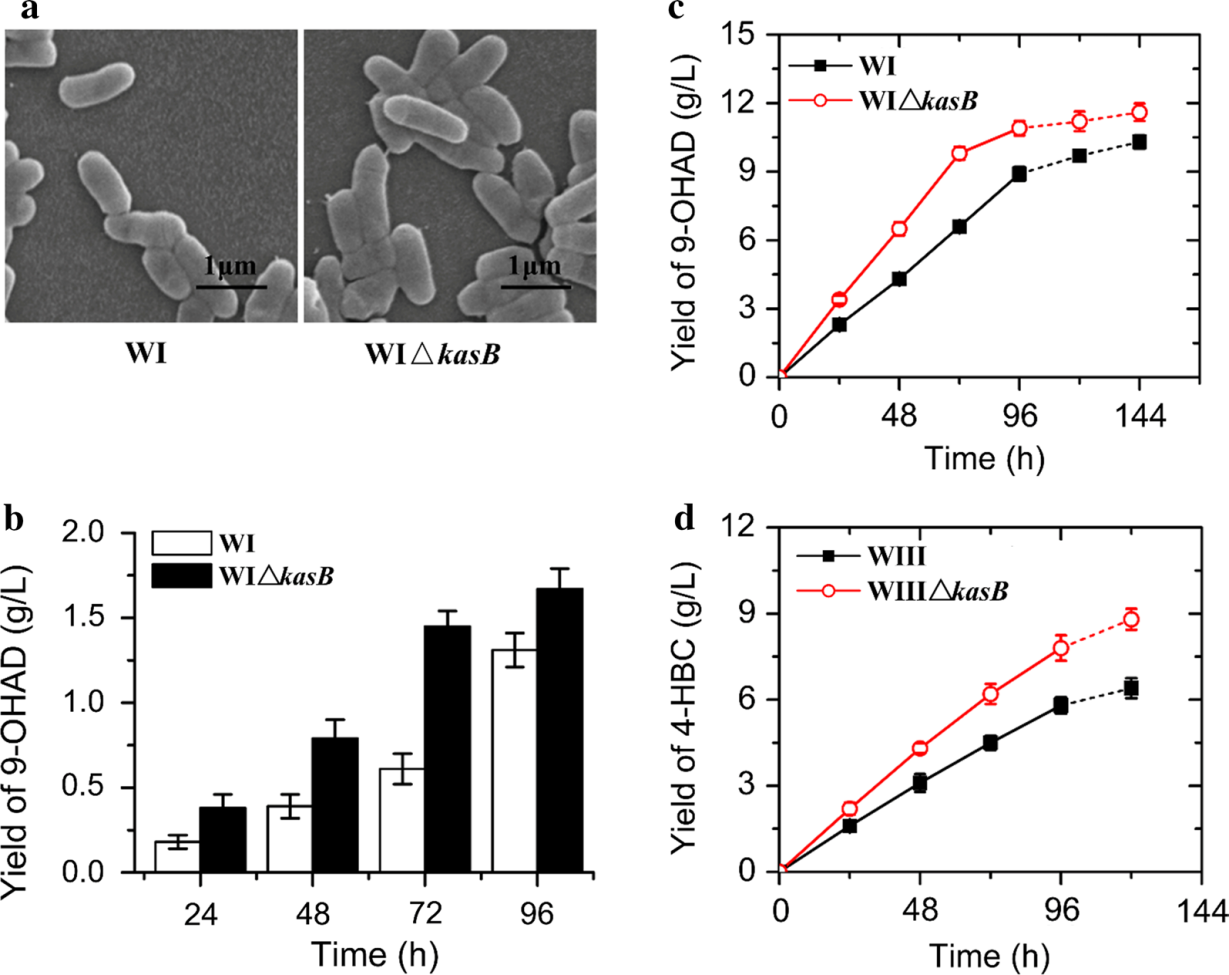
Enhancement of the 9-OHAD productivity in *M. neoaurum*. **a** Cell morphologies of the engineered mutant strains revealed by a scanning electron microscope. The cell morphology of the WIΔ*kasB* stain showed no obvious defects compared to that of its parental strain WI. **b** Assessment of 9-OHAD yield for the deficiency of *kasB*. Quantitative analyses of the 9-OHAD yield in the vegetative cell transformation of 5 g/L phytosterols. **c** Determination of the C19 intermediate 9-OHAD productivity in the constructed 9-OHAD-producing strain WIΔ*kasB* by a resting cell system containing 20 g/L of phytosterols. **d** Measurement of the C22 intermediate 4-HBC productivity in the engineered producer WIIIΔ*kasB* by resting cell conversion in the presence of 20 g/L phytosterols

To determine the effect of altered MAMEs and permeability on the production of steroidal intermediates, the transformation phenotype of the 9-OHAD-producing strain WI and WIΔ*kasB* was determined. The result showed that the growth speed of the mutant strain WIΔ*kasB* was not changed obviously under the sterol-free culture conditions (Additional file 2: Figure S5). In addition, the cell morphology of mutant strain was unaffected apparently (Fig. 4a). This phenomenon was different with the deletion of *kasB* in *M. tuberculosis* [16]. These results indicated that the *kasB* was possibly not the sole functional enzyme involved in the specific elongation step of mero-MAs in *M. neoaurum* ATCC 25795. Despite the deficiency of *kasB*, the stability of cellular structure could be still maintained in *M. neoaurum*. In view of the enhanced uptake of sterols resulted from the altered cell permeability, the accumulation capability of target steroids was preliminary analyzed. The vegetative cell transformation led to a remarkably increased 9-OHAD yield in the WIΔ*kasB* strain compared to its parental strain (Fig. 4b). The deletion of *kasB* increased the target steroid by 137.7% from 0.61 to 1.45 g/L after 72-h conversion. However, the increase precipitously declined to 28% after 96-h of biotransformation.

Next, a resting cell bioconversion system widely applied in the industry was used to further assess the enhancement effect of C19 steroid intermediate 9-OHAD generated by the *kasB* deletion (Fig. 4c). The highest increase was detected in WIΔ*kasB* strain after 72-h transformation with the production of 9.8 g/L, which was 48.5% higher than that of its parental WI strain (6.6 g/L). Ultimately, the WIΔ*kasB* strain yielded 10.9 g/L 9-OHAD with a molar yield of 69.5%, whereas its parental strain WI only produced 8.9 g/L with a molar yield of 56.7%. In addition, if the bioconversion time was extended by 48 h, the 9-OHAD production of WI strain would increase to about 10.3 g/L, which was still lower than that of the WIΔ*kasB* strain. In other words, the modification of *kasB* gene shortened the conversion time by more than 33%. The screened *kasB* stably remodeled the cell wall mycolic acid component, thus resulting in an increase of 22.5% in the production of C19 steroidal 9-OHAD.

The enhancement effect of *kasB* deficiency had been tested in another typical C22 steroidal intermediate 4-HBC producing strain WIII [7]. Similarly, an obvious improvement in the target intermediate was detected in the vegetative WIIIΔ*kasB* cell (Additional file 2: Figure S6), indicating that the strategy of disrupting the mycolic acid synthesis might be efficient for the stable evolution towards target steroidal producer. Accordingly, the assessment of resting cells showed that the 4-HBC production in the WIIIΔ*kasB* strain was increased by 34.5% from 5.8 g/L to 7.8 g/L after 96-h conversion (Fig. 4d). In addition, the 4-HBC yield was improved by 37.5% from 6.4 to 8.8 g/L after 120-h biotransformation [2]. Thus, the modification of *kasB* is highly effective for the self-enhancement of steroid intermediate conversion in *M. neoaurum*.

## Conclusions

This study aimed to develop a gentle and stable self-excitation strategy of steroid intermediate conversion by the disruption of cell wall components in mycobacterial cells. To understand the important role of MAs in cell permeability related to the uptake of sterol substrate, the dispensable genes of MA synthesis in *M. neoaurum* were deleted respectively. The modification of *kasB* showed a striking increase in sterol conversion rate, indicating a meaningful change in the cell wall mycolic acids. The deficiency of the screened *kasB* gene significantly changed the cell wall permeability by altering the constitution of MAMEs and shortening the length of mycolic acids in the cell wall, thus resulting in an efficient self-enhancement of steroidal intermediate conversion.

## Supplementary information

**Additional file 1: Table S1.** Plasmids used in this study. **Table S2.** Primers used in this study. **Table S3.** Identification and annotation of the mycolic acid synthesis related genes. **Table S4.** Comparisons of *kasB* region in mycobacteria.

**Additional file 2: Figure S1.** Comparison of the localization of *kasB* homologous gene in mycobacteria. **Figure S2.** In-frame deletion of *kasB* in *M. neoaurum* ATCC 25795. **Figure S3.** Absolute intensity of the mycolate in *M. neoaurum*. **Figure S4.** MALDI-TOF mass spectra of the keto-MAMEs of *M. neoaurum* strains. **Figure S5.** Growth curve of the *kasB* mutant strain. **Figure S6.** Assessment of 4-HBC production for the deletion of *kasB* in the typical 4-HBC-producing strain MnΔ*kshA*Δ*hsd4A*Δ*kstD1*Δ*kstD2*Δ*kstD3* (WIII).

## Data Availability

All data generated or analyzed during this study are included in this published article and its additional files.

## Abbreviations

KasB: β-Ketoacyl-acyl carrier protein synthase
MA: Mycolic acids
9-OHAD: 9*α*-Hydroxy-4-androstene-3,17-dione
4-HBC: 22-Hydroxy-23,24-bisnorchol-4-ene-3-one
AD: Androst-4-ene-3,17-dione
BD: Boldenone
MAMEs-AG-PG: Mycolyl-arabinogalactan-peptidoglycan
FASII: Fatty acid synthase II
FDA: Fluorescein diacetate
TMM: Trehalose monomycolate
TDM: Trehalose dimycolate
MAMEs: Mycolic acid methyl esters
LB: Luria–Bertani
MM: Minimal medium
HPLC: High performance liquid chromatography
GC: Gas chromatography
FAS-I: Fatty acid synthase I
FabD: Malonyl CoA-acyl carrier protein (ACP) transacylase
FabH: β-Ketoacyl-ACP synthase III
MabA: β-Ketoacyl-ACP reductase
HadABC: β-Hydroxyacyl-ACP dehydratase subunits A, B and C
InhA: Enoyl-ACP reductase
KasA: β-Ketoacyl-ACP synthase 1
PcaA: Proximal cyclopropanation of alpha-MAs enzyme
MmaA1-4: Methyl mycolic acid synthase
CmaA2: Cyclopropyl mycolic acid synthase
AccD4: Propanoyl-CoA carbon dioxide ligase
AccD5: Propionyl-CoA carboxylase
FadD32: Long-chain-fatty-acid-AMP synthetase
Pks13: Polyketide synthase
