# Supplementary material for: Enhancing the bioconversion of phytosterols to steroidal intermediates by the deficiency of kasB in the cell wall synthesis of Mycobacterium neoaurum
Source: Microb Cell Fact. 2020 Mar 30;19:80. doi: 10.1186/s12934-020-01335-y (PMC7106593; doi:10.1186/s12934-020-01335-y)
Supplement: Supplementary file 1 — Additional file 1: Table S1. Plasmids used in this study. Table S2. Primers used in this study. Table S3. Identification and annotation of the mycolic acid synthesis related genes. Table S4. Comparisons of kasB region in mycobacteria. [file 12934_2020_1335_MOESM1_ESM.docx]

# Additional file 1

# Table S1 Plasmids used in this study

| **Name** | **Description** | **Source** |
| --- | --- | --- |
| p2NIL | Vector for constructing homologous arms of mycobacteria, *Kan^R^* | [[1](#_ENREF_1)] |
| p2N-*hadA* | p2NIL carrying two homologous arms of *hadA*, *Kan^R^* | This study |
| p2N-*hadC* | p2NIL carrying two homologous arms of *hadC*, *Kan^R^* | This study |
| p2N-*kasB* | p2NIL carrying two homologous arms of *kasB*, *Kan^R^* | This study |
| p2N-*mmaA_N_* | p2NIL carrying two homologous arms of *mmaA_N_*, *Kan^R^* | This study |
| p2N-*pks13* | p2NIL carrying two homologous arms of *pks13*, *Kan^R^* | This study |
| pGOAL19 | *Hyg* Pag85-*lacZ* P*_hsp60_-sac*B, *PacⅠ*cassette vector, *Amp^R^* | [[1](#_ENREF_1)] |
| p19-*hadA* | p2NIL-derived with selection cassette from pGOAL19 for deletion of *hadA* in mycobacteria | This study |
| p19-*hadC* | p2NIL-derived with selection cassette from pGOAL19 for deletion of *hadC* in mycobacteria | This study |
| p19-*kasB* | p2NIL-derived with selection cassette from pGOAL19 for deletion of *kasB* in mycobacteria | This study |
| p19-*mmaA_N_* | p2NIL-derived with selection cassette from pGOAL19 for deletion of *mmaA_N_* in mycobacteria | This study |
| p19-*pks13* | p2NIL-derived with selection cassette from pGOAL19 for deletion of *pks13* in mycobacteria | This study |
| pMV261 | Shuttle vector of *M. neoaurum* and *E. coli* carrying the heat shock (*hsp60*) promoter, *Kan^R^* | [[2](#_ENREF_2)] |
| p261-*kasB* | Recombinant pMV261, for overexpression of *kasB* in mycobacteria | This study |
| pMV306 | Integration vector with single copy in *M. neoaurum*, without promoter, *Kan^R^* | [[3](#_ENREF_3)] |
| p306-*kasB* | pMV306-P*hsp60*-*kasB*, integrative into mycobacterial chromosomal DNA | This study |

**Table S****2** Primers used in this study

| **Primers** | **Description** |
| --- | --- |
| **For gene deletion** |  |
| D-*hadA*-UF & UR | TATCaagcttACCGACATCCGCCGGCTCACGA & TATCgctagcGCTCGGCGGCGACAGCTT |
| D-*hadA*-DF & UR | TATCgctagcTCGATTCGGTGCGTCAGGCG & TATCggatccGATTGACGAACCGACGGACAGC |
| D-*hadC*-UF & UR | GCGTaagcttCCAAGAAGAACCGTCGGAACGA & ATAGgctagcCAACCGGTCGTGACCCAGCT |
| D-*hadC*-DF & UR | TAGTgctagcTTCAACGCCGATATCGTCGTGA & ATACggtaccTTCTCGTAACCGGCGTACGAGT |
| D-*kasB*-UF & DR | ATACaagcttTCGTGATCGGCACCGGGCTC & GACTgaattcTACTCCTCGACGAACGGGTC |
| D-*kasB*-DF & DR | AGACgaattcCGAGATCGACCTGGACGTGG & TATCggatccAAGGCATCGCACAACCGCAC |
| D-*mmaA1*-UF& UR | ACTGaagcttACCGAAGAGGGCCAGTTCCA & TCTGgaattcGATGTCGTAGATGGACTGCGA |
| D-*mmaA1*-DF & DR | ATCAgaattcACGCCCGCACGCTGGACAT & TATCggatccGTTGTGAGCCAGGACAACAC |
| D-*mmaA2*-UF & UR | ATACaagcttGCTGCCCTCAGCTCCGCGCT & ATACgaattcTGGGCCGCGGCGCTGGAG |
| D-*mmaA2*-DF & DR | ATGAgaattcCGCGAGCTGGGCCTCTTCCAG & ATACggatccGCGAAACCGATGGCCTGGAG |
| D-*mmaA3*-UF & UR | CGACaagcttAGCTGCTCTGGATGGTCATCC & ATACgaattcCGCGCACGCTGGATCTGTGG |
| D-*mmaA3*-DF & DR | AGACgaattcTCTTGGCCAACTGCGCCTCG & AGACggtaccACTGGACATCGTCTACGACG |
| D-*mmaA4*-UF & UR | TATCaagcttACGGTTGCGGCCACCGCG & GCGAccatggAATCACTACATCAAGACCCTC |
| D-*mmaA4*-DF & DR | ACTGccatggACGCCGAAGAAATCATCGGAC & ATCTggatccCAGAAAGCCCACGTGGAAGC |
| D-*pks13*-UF & UR | GCGCaagcttCATCGTCGAGGTCGACGCCGATTCG & GCGAgaattcGACGCCGGTCAGATCCTCGATATCG |
| D-*pks13*-DF & DR | GCGCgaattcCACGATGACGCCATCGTCTTCGAAC & TATAgcggccgcAGCAGGATCTCGTGCATGTCGTAAC |
| **For gene complement** |  |
| C-p261-F & R | TAGGCGAGTGCTAAGAATAACGTT & TCGTTTTATTTGATGCCTGGCAGT |
| C-*kasB*-F & R | TCTGgaattcGTGGGGAACGGCTTGAACAAG & AGTGaagcttTCAGTACTTTCCGAAGGTGAG |

**Table S3** Identification and annotation of the mycolic acid synthesis related genes

| **Gene ID** | **Log_2_[(Mn+C) vs Mn]** | **P−Value** | **Log_2_[(Mn**Δ***k1*+C) vs (Mn+C)]** | **P−Value** | **Accession** | **Sequence Description** | **Annotation** |
| --- | --- | --- | --- | --- | --- | --- | --- |
| *Mn_3604* | 0.98 | 2.53E-76 | 2.29 | 0 | gi\|433649062 | NZ_JMDW01000005.1; 309323…318553 − | fatty acid synthase-I |
| *Mn_1414* | 1.86 | 9.48E-146 | 0.37 | 2.19E-254 | gi\|407984122 | NZ_JMDW01000013.1; 174784…175647 + | malonyl CoA-acyl carrier protein transacylase, FabD |
| *Mn_4227* | 1.29 | 5.92E-11 | 1.25 | 2.31E-61 | gi\|404419785 | NZ_JMDW01000008.1; 293874…294350 + | (3R)-hydroxyacyl-ACP dehydratase subunit, HadA |
| *Mn_4228* | 1.92 | 4.17E-09 | 1.30 | 1.31E-37 | gi\|404419786 | NZ_JMDW01000008.1; 294337…294765 + | (3R)-hydroxyacyl-ACP dehydratase subunit, HadB |
| *Mn_4229* | 0.28 | 0.444554 | 1.97 | 4.94E-20 | gi\|118471308 | NZ_JMDW01000008.1; 294769…295275 + | (3R)-hydroxyacyl-ACP dehydratase subunit, HadC |
| *Mn_0489* | −0.78 | 2.35E-10 | 1.16 | 7.03E-05 | gi\|108799424 | NZ_JMDW01000010.1; 357277…358086 − | NADH-dependent enoyl-[ACP] reductase, InhA |
| *Mn_1416* | 0.32 | 3.8E-09 | 1.20 | 5.75E-277 | gi\|375141495 | NZ_JMDW01000013.1; 176055…177263 + | 3-oxoacyl-[acyl-carrier-protein] synthase 1, KasA |
| *Mn_1417* | 0.31 | 0 | 0.90 | 1.95E-122 | gi\|108800333 | NZ_JMDW01000013.1; 177370…178587 + | 3-oxoacyl-[acyl-carrier-protein] synthase 2, KasB |
| *Mn_4061* | 2.18 | 2.06E-120 | −0.16 | 5.50E-106 | gi\|404423367 | NZ_JMDW01000008.1; 138276…139148 + | methyl mycolic acid synthase 1, MmaA1 |
| *Mn_4052* | 1.54 | 3.86E-233 | −0.09 | 8.03E-216 | gi\|333990666 | NZ_JMDW01000008.1; 129019…129894 − | methyl mycolic acid synthase 2, MmaA2 |
| *Mn_4247* | 1.60 | 1.84E-28 | −0.46 | 2.63E-14 | gi\|374613407 | NZ_JMDW01000008.1; 325934:326845 − | methyl mycolic acid synthase 3, MmaA3 |
| *Mn_4246* | 1.39 | 2.82E-64 | 0.45 | 1.79E-138 | gi\|108797925 | NZ_JMDW01000008.1; 324556…325503 − | methyl mycolic acid synthase 4, MmaA4 |
| *Mn_2637* | 0.03 | 0.74678 | 1.03 | 8.17E-48 | gi\|433650743 | NZ_JMDW01000024.1; 38973…40547 + | propanoyl-CoA carbon dioxide ligase, AccD4 |
| *Mn_4683* | 2.26 | 1.73E-191 | 0.41 | 0 | gi\|404420268 | NZ_JMDW01000008.1; 788607…790235 − | propionyl-CoA carboxylase subunit beta, AccD5 |
| *Mn_2634* | 1.33 | 1.19E-128 | 0.84 | 0 | gi\|441217120 | NZ_JMDW01000024.1; 31599…33488 + | long-chain-fatty-acid-AMP synthetase, FadD32 |
| *Mn_2636* | −0.47 | 6.62E-19 | 1.50 | 4.90E-148 | gi\|118469591 | NZ_JMDW01000024.1; 33643…38976 + | polyketide synthase, Pks13 |

**Table S4** Comparisons of *kasB* region in mycobacteria

| **Mn^a^** | **MnV^b^ (ID)** | **Ms^c^ (ID)** | **Mt^d^** | **Annotation** |
| --- | --- | --- | --- | --- |
| *Mn_1414* (*fabD*) | *D174_18295* | *MSMEG_4325* | *fabD* | Malonyl CoA-acyl carrier protein transacylase |
| *Mn_1415* (*acpM*) | *D174_18300* | *MSMEG_4326* | *acpM* | Meromycolate extension acyl carrier protein |
| *Mn_1416* (*kasA*) | *D174_18305* | *MSMEG_4327* | *kasA* | 3-oxoacyl-[acyl-carrier-protein] synthase 1 |
| *kasB* | *D174_18310* | *MSMEG_4328* | *kasB* | 3-oxoacyl-[acyl-carrier-protein] synthase 2 |
| *Mn_1418* (*accD6*) | *D174_18315* | *MSMEG_4329* | *accD6* | acetyl-CoA carboxylase subunit beta |
| *Mn_1419* | *D174_18320* | *MSMEG_4330* | *Rv2248* | — |

Note:

1. ^a^ Mn, *M. neoaurum* ATCC 25795; ^b^ MnV, *M. neoaurum* VKM Ac-1815D; ^c^ Ms, *M. smegmatis* mc2 155; ^d^ Mt, *M. tuberculosis* H37Rv.

# References

[1] Gordhan BG, Parish T. Gene replacement using pretreated DNA. Methods Mol Med. 2001;54:77−92.

[2] Stover CK, de la Cruz VF, Fuerst TR, Burlein JE, Benson LA, Bennett LT, Bansal GP, Young JF, Lee MH, Hatfull GF, et al. New use of BCG for recombinant vaccines. Nature. 1991;351:456−60.

[3] Stover CK, de la Cruz VF, Bansal GP, Hanson MS, Fuerst TR, Jacobs Jr WR, Bloom BR. Use of recombinant BCG as a vaccine delivery vehicle. Adv Exp Med Biol. 1992;327:175−82.
