# Supplementary material for: Enhancing the bioconversion of phytosterols to steroidal intermediates by the deficiency of kasB in the cell wall synthesis of Mycobacterium neoaurum
Source: Microb Cell Fact. 2020 Mar 30;19:80. doi: 10.1186/s12934-020-01335-y (PMC7106593; doi:10.1186/s12934-020-01335-y)
Supplement: Supplementary file 2 — Additional file 2: Figure S1. Comparison of the localization of kasB homologous gene in mycobacteria. Figure S2. In-frame deletion of kasB in M. neoaurum ATCC 25795. Figure S3. Absolute intensity of the mycolate in M. neoaurum. Figure S4. MALDI-TOF mass spectra of the keto-MAMEs of M. neoaurum strains. Figure S5. Growth curve of the kasB mutant strain. Figure S6. Assessment of 4-HBC production for the deletion of kasB in the typical 4-HBC-producing strain MnΔkshAΔhsd4AΔkstD1ΔkstD2ΔkstD3 (WIII). [file 12934_2020_1335_MOESM2_ESM.docx]

# Additional file 2


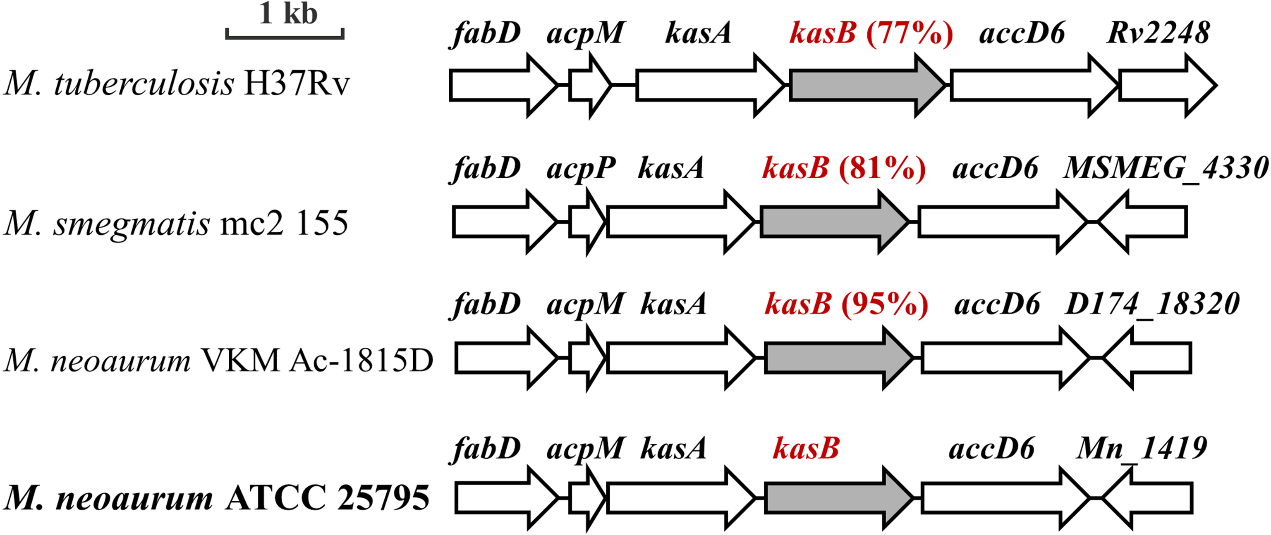


**Fig. S1** Comparison of the localization of *kasB* homologous gene in mycobacteria. The percentages respect the sequence identity in *M. neoaurum* ATCC 25795 with the homologues from the other three mycobacteria.

**
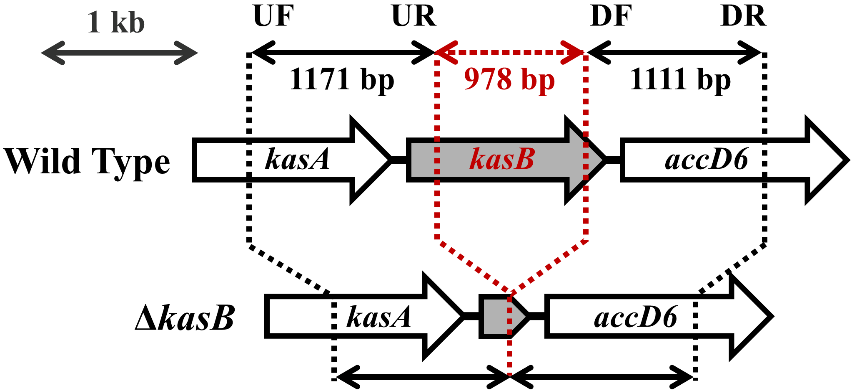
**

**Fig. S2** In-frame deletion of *kasB* in *M. neoaurum* ATCC 25795. A 1,171-bp upstream fragment and a 1,111-bp downstream fragment were designed as allelic recombination arms.





**Fig. S3** Absolute intensity of the mycolate in *M. neoaurum*.


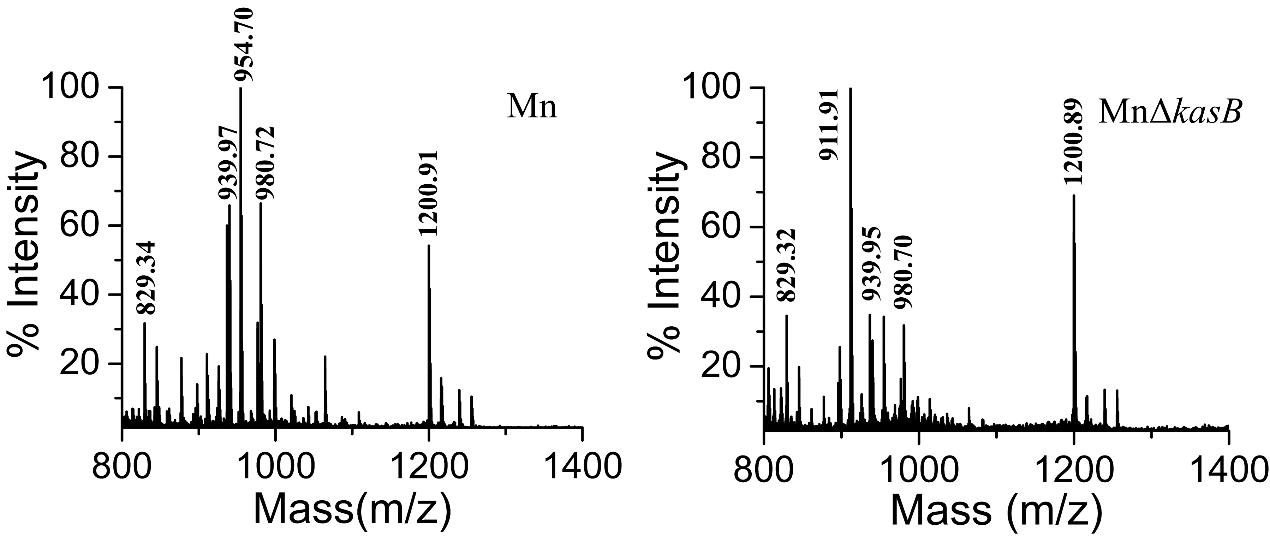


**Fig. S4** MALDI-TOF mass spectra of the keto-MAMEs of *M. neoaurum* strains.


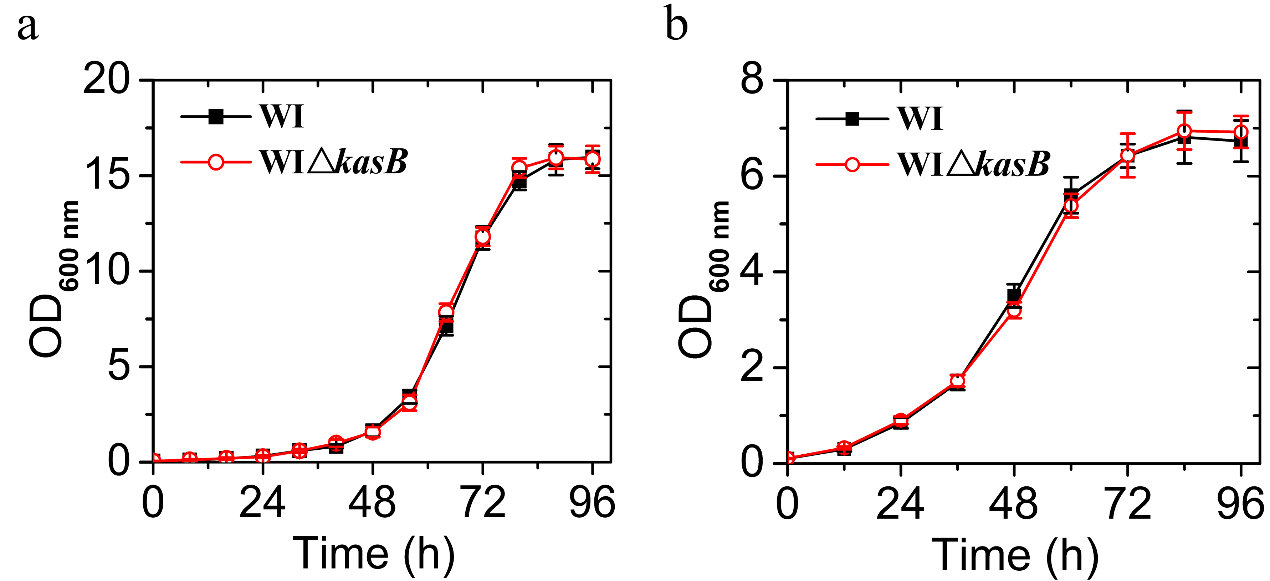


**Fig. S5** Growth curve of the *kasB* mutant strain. K*asB* was deleted in the previously constructed 9-OHAD-producing strain MnΔ*kstD1*Δ*kstD2*Δ*kstD3* (WI). **a** Strains were cultivated in MYC01 medium. **b** Strains were cultured in MYC02 medium.

**

**

**Fig. S6** Assessment of 4-HBC production for the deletion of *kasB* in the typical 4-HBC-producing strain MnΔ*kshA*Δ*hsd4A*Δ*kstD1*Δ*kstD2*Δ*kstD3* (WIII) in the vegetative cell transformation of 4 g/L phytosterols.
